# Anti-centromere antibody-seropositive Sjögren's syndrome differs from conventional subgroup in clinical and pathological study

**DOI:** 10.1186/1471-2474-11-140

**Published:** 2010-07-01

**Authors:** Hideki Nakamura, Atsushi Kawakami, Tomayoshi Hayashi, Naoki Iwamoto, Akitomo Okada, Mami Tamai, Satoshi Yamasaki, Hiroaki Ida, Katsumi Eguchi

**Affiliations:** 1 Unit of Translational Medicine, Department of Immunology and Rheumatology, Nagasaki University Graduate School of Biomedical Sciences, 1-7-1 Sakamoto, Nagasaki City, Nagasaki 852-8501, Japan; 2 Department of Pathology, Nagasaki University Hospital, 1-7-1 Sakamoto, Nagasaki City, Nagasaki 852-8501, Japan

## Abstract

**Background:**

To clarify the clinicopathological characteristics of primary Sjögren's syndrome (pSS) with anti-centromere antibody (ACA).

**Methods:**

Characteristics of 14 patients of pSS with ACA were evaluated. All patients were anti-SS-A/Ro and SS-B/La antibodies negative (ACA+ group) without sclerodactyly. The prevalence of Raynaud's phenomenon (RP), titer of IgG and focus score (FS) in the minor salivary glands (MSGs) were determined. Quantification analysis of Azan Mallory staining was performed to detect collagenous fiber. Forty eight patients in whom ACA was absent were chosen as the conventional (ACA-) pSS group.

**Results:**

Prevalence of ACA+ SS patients was 14 out of 129 (10.85%) pSS patients. RP was observed in 61.5% of the patients with ACA. The level of IgG in the ACA+ group was significantly lower than that of the ACA- group (p = 0.018). Statistical difference was also found in the FS of MSGs from the ACA+ group (1.4 ± 1.0) as compared with the ACA- group (2.3 ± 1.6) (p = 0.035). In contrast, the amount of fibrous tissue was much higher in the ACA+ group (65052.2 ± 14520.6 μm^2 ^versus 26251.3 ± 14249.8 μm^2 ^) (p = 1.3 × 10^-12^).

**Conclusions:**

Low cellular infiltration but with an increase in fibrous tissues may explain the clinical feature of a high prevalence of RP and normal IgG concentration in ACA+ pSS.

## Background

Primary Sjögren's syndrome (pSS) is characterized by sicca symptoms and various extraglandular manifestations that are usually accompanied by autoantibodies, especially anti-SS-A/Ro and SS-B/La antibodies (Abs) [1]. Except for anti-SS-A/Ro or SS-B/La antibodies, some autoantibodies including anti-α-fodrin antibody or anti-type 3 muscarinic acetylcholine receptor antibodies are found in pSS [2,3]. Anti-centromere antibody (ACA) recognizes several centromere antigens of human chromosomes (CENP), in which CENP-B is a highly conserved mammalian protein and is used for detection by enzyme-linked immunosorbent assay (ELISA) [4]. Recent studies [5,6] have demonstrated that ACA is also associated with pSS. Although the prevalence of anti-SS-A/Ro or SS-B/La Abs in pSS is 60-70%, ACA is reported to appear in 16-26% of patients with pSS. Some reports [7] have shown the clinical characteristics of ACA-positive (ACA+) pSS, and a high frequency of Raynaud' s phenomenon (RP) has been repeatedly reported. In addition to the low prevalence of RP, Katano et al [8] reported a low titer of IgG in an ACA+ and anti-SS-A/Ro antibody-negative pSS population.

The appearance of ACA is commonly described in CREST syndrome (calcinosis, RP, esophageal dysmotility, sclerodactyly, telangiectacia) or the limited type of systemic sclerosis (SSc) [9]. Although the presence of ACA is considered to be related to fibrosis of various organs, no relationship between fibrosis and ACA in pSS is reported. In the present study, we found the predominance of fibrotic change of minor salivary glands (MSG) histologically in ACA+ pSS patients. Interestingly, cellular infiltration was less prevalent in ACA+ pSS patients. These differences are suggested to lead to the clinical characteristics of ACA+ pSS.

## Methods

### Patients

Fourteen pSS patients with ACA were included in the present study (ACA+ group). The classification of pSS was determined by the revised criteria for the diagnosis of pSS, as proposed by the American-European Consensus group (AECG) [10]. All ACA+ pSS patients without sclerodactyly are also seronegative toward anti-SS-A/Ro Ab or anti-SS-B/La Ab in this study. All ACA+ pSS patients were female, and their other clinical and serological features are described in Table 1. The measurement of anti-SS-A/Ro Ab, anti-SS-B/La Ab (Mesacup SS-A/Ro test and SS-B/La Test; Medical & Biological Laboratories, Nagoya, Japan), serum IgG concentration and ACA (ELISA kit, Mesacup-2 test CENP-B; Medical & Biological Laboratories, Nagoya, Japan) was performed as described previously [5]. Serum IgG concentration was measured by a nephelometric immunoassay. Two out of 14 ACA+ pSS patients complained fatigability and no hematological disorders such as malignant lymphoma was observed in the medical records. With regard to usage of medications, 2 patients used pilocarpine hydrochloride, other 2 patients used cevimeline hydrochloride hydrate and 1 patient used synthetic saliva spray as oral medication. Regarding ophthalmic drop, 3 patients used artificial tear drop and 1 patient used cyanocobalamin for asthenopia.

**Table 1:** Background of ACA+ and ACA- SS patients in this study

	ACA (+)	ACA (-)	P value
N (M/F)	14 (0/14)	48 (1/47)	0.59
Age	57.4 ± 9.6	58.3 ± 13.2	0.82
Follow-up period (year)^¶^	6.6 ± 5.6	4.5 ± 4.6	0.16
Raynaud's phenomenon	8/13 (61.5%)	4/48 (8.3%)	1.86 × 10-5
anti-SS-A/Ro Ab or anti-SS-B/La Ab	0/14 (0.0%)	37/48 (77.1%)	2.30 × 10-7
IgG (mg/dl)	1530.2 ± 267.1	2056.0 ± 730.2	0.018
Average of FS	1.4 ± 1.0	2.3 ± 1.6	0.035

Clinical characteristics of the ACA-seropositive and non-ACA Sjögren's syndrome (SS) patients are shown. The differences were calculated using Student's *t *test and the Chi-square test. (*; p < 0.05; statistically significant). The differences found regarded the prevalence of Raynaud's phenomenon, the level of IgG and the average of FS.

^¶^Follow-up period is duration from point of diagnosis. ACA; anti-centromere antibody, FS; focus score

For comparison to ACA+ pSS patients, 48 pSS patients in the absence of ACA were selected to be the conventional (ACA-) group for pathological study. The prevalence of anti-SS-A/Ro Ab or anti-SS-B/La Ab in the conventional group was 77.1% as listed in Table 1.

### Biopsy of labial salivary glands

Labial salivary gland biopsy was performed for all of the pSS patients. Informed consent for the usage of samples obtained by the biopsy was obtained from all the participating patients as of the commencement of the study, and the study was conducted with the approval of the human ethical committee of our institution. The severity of mononuclear cell infiltration was determined using the classifications of Chisholm & Mason [11], whereas the focus score (FS) was also examined using a classification determined by Greenspan [12].

### Azan Mallory staining of labial salivary glands

Formalin-fixed, paraffin-embedded sections (3-μm thick) from labial salivary glands of 14 ACA+ pSS patients and 48 ACA- pSS patients were cut and mounted onto glass slides precoated with aminopropyltriethoxysilane (APS). For the detection of collagenous fiber in MSG, Azan Mallory staining was performed. To calculate the area of the blue-stained lesion obtained by Azan Mallory staining, WinROOF software (Mitani Corporation, Fukui, Japan) was employed. Briefly, the software captured the blue signal on the samples, and the total area was automatically calculated by WinROOF.

### Statistical analysis

Serological data, FS and the calculated blue signal area in MSGs of both groups were compared by Student's *t *test or the Chi square test. A *P *value less than 0.05 was considered statistically significant.

## Results

### Clinical manifestations of ACA+ pSS patients

In our institution, the prevalence of ACA+ pSS patients was 14 out of 129 (10.85%) pSS patients who strictly met AECG classification criteria in our medical records, although the entire 129 pSS patients were listed with or without examination for labial salivary biopsy.

The basic information of the ACA+ pSS patients is indicated in Table 1. All of the registered patients were females, and no statistically significant difference was observed regarding gender and age between the 14 ACA+ patients (57.4 ± 9.6) and the 48 ACA- patients (58.3 ± 13.2) (p = 0.82). There was no statistical difference in follow-up period from point of diagnosis between ACA+ and ACA- pSS groups. ACA- patients showed either xerophtalmia (56.3%) or xerostomia (72.9%) with 85.7% positivity in the Saxon test. In addition, these 3 parameters showed no statistical significance between ACA- and ACA+ pSS patients (P value determined by Chi-square test was 0.12, 0.59 and 0.92, respectively). The prevalence of RP in the ACA+ group was 8/13 (61.5%). Serum IgG was 1530.2 ± 267.1 mg/dl (normal range; 870-1700), which was within the normal limit. The serum IgG from the ACA- group was 2056.0 ± 730.2, which showed a statistically significant difference from that of the ACA+ group (p = 0.018). Furthermore, the serum IgG from ACA- pSS group without anti-SS-A/Ro or SS-B/La Abs was 1615.7 ± 361.5, which was statistically similar to that of ACA+ pSS group (p = 0.63). We also obtained quantitative results regarding ACA by ELISA from 13 SS patients; these results showed that the titer of ACA of these patients was 157.0 ± 33.9 (normal range; <16 index). The results of MSG biopsy showed 2 patients in grade 2, 7 patients in grade 3 and 5 patients in grade 4 according to the Chisholm & Mason classification with the FS of 1.4 ± 1.0. Compared to the FS of the 48 patients in the ACA- group (2.3 ± 1.6), mononuclear cell infiltration was significantly lower in the ACA+ pSS patients (p = 0.035).

### Increment of collagenous fiber in ACA+ pSS patients

**Figure 1:**
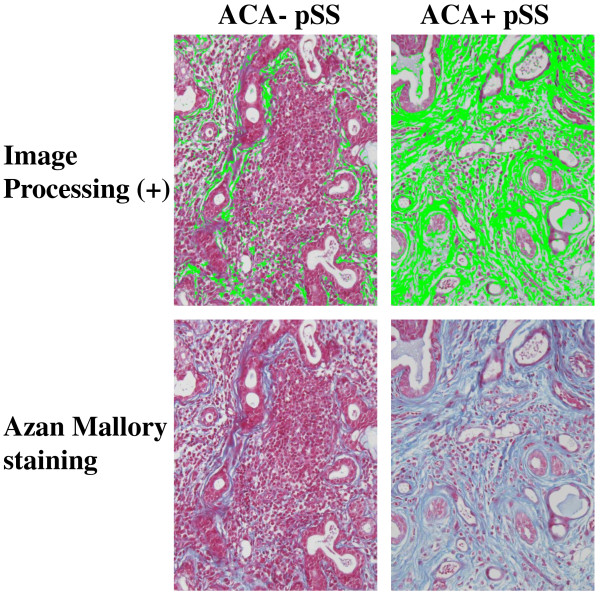
**Azan Mallory staining of anti-centromere antibody (ACA) + and ACA- patients with Sjögren's syndrome (SS)**. To detect the existence of collagenous fiber in minor salivary glands in SS patients, Azan Mallory staining was performed for all patients. The lower panels show Azan Mallory staining results of ACA+ and ACA- SS patients. To clarify the amount of collagenous fiber, the image analyzer WinROOF was employed. The green signal in the upper panels shows the captured form of blue staining produced by Azan Mallory staining. (Original magnification × 200).

**Figure 2:**
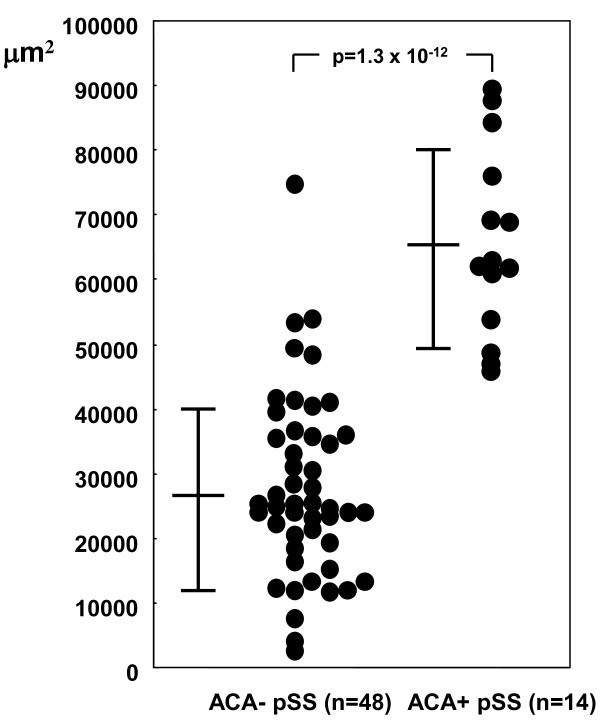
**Quantification of captured signal in minor salivary glands in anti-centromere antibody (ACA) + and ACA- patients with Sjögren's syndrome (SS)**. The captured green signal from 14 ACA+ and 48 ACA- SS patients was calculated by WinROOF software under certain conditions. The figures represent actual measurements in units of μm^2 ^under a fixed magnification ratio of the optical microscope (×200). A scatter plot of the processed data is shown. Quantification results from the patients with and without ACA showed 65052.2 ± 14520.6 μm^2 ^and 26251.3 ± 14249.8 μm^2 ^with significance (p = 1.3 × 10^-12^). Student's *t *test was used for statistical evaluation. A *P *value less than 0.05 was considered statistically significant.

To evaluate the amount of collagenous fiber in MSG, lesions stained blue by Azan Mallory staining were captured by an image analysis system. The MSG of the ACA+ group showed large amounts of collagenous fiber compared to that in the ACA- group (Figure 1). Under certain conditions, the captured signal area of MSG from both groups was calculated by WinROOF software. The calculated areas in the ACA+ group and the ACA- group were 65052.2 ± 14520.6 μm^2 ^and 26251.3 ± 14249.8 μm^2 ^with statistical significance, respectively (p = 1.3 × 10^-12^) (Figure 2).

## Discussion

Although ACA is known to appear in CREST syndrome or limited cutaneous SSc patients with RP, its significance in pSS remains to be clarified precisely. With regard to the prevalence of ACA in pSS patients, Salliot C et al [13] previously described as 4.7% in 212 pSS patients in France. Prevalence in our data (10.85%) was statistically higher than that of their data (p = 0.032 by chi-square test) despite the same measurement of antibodies against CENP-B. It might derive from genetic background or difference of ELISA kit. Our present data confirmed the previous observation that ACA+ pSS has a high prevalence of RP despite normal IgG, which is different from ACA- pSS, which is seropositive toward anti-SS-A/Ro Ab or anti-SS-B/La Ab [5,8]. However, we should note that the serum IgG from ACA- pSS group without anti-SS-A/Ro or SS-B/La Abs and ACA+ pSS group was within normal limit without statistical significance. These observations suggest normal IgG might be associated with negativity of anti-SS-A/Ro or SS-B/La Abs rather than ACA. Since there was no statistical difference with regard to follow-up period between ACA+ and ACA- pSS groups in this study, we suggest that the high prevalence and normal IgG in ACA+ pSS is specific for this disease subset. It is speculated that fibrotic change might be dominant in ACA+ pSS, and we have revealed for the first time using quantitative analysis of Azan Mallory staining that collagenous fiber is accumulated in the MSG of ACA+ pSS patients. In addition, a novel finding was made that there is less mononuclear cell infiltration as determined by FS in ACA+ pSS patients as compared with conventional pSS patients. In rheumatoid arthritis, Oosterhout *et al *recently found the histological difference based on the presence or absence of anti-cyclic citrullinated peptide (CCP) antibodies in the synovial tissues that synovial tissues from anti-CCP antibodies+ subjects contain more cellular components with less fibrotic change as compared with those from anti-CCP antibodies - subjects [14]. This finding suggests that the serologic status may reflect the histologic characteristics in autoimmune diseases, and our present report is the first one involving patients with pSS. In ACA+ pSS patients, a lower FS appears to be associated with normal serum IgG whereas prominent collagenous fiber accumulation correlates with a high prevalence of RP. More recently, Bournia VK et al [15] reported that hypergammaglobulinemia was characteristic of not ACA+ pSS but ACA-SS subgroup, which suggests ACA+ pSS subgroup forms intermediate entity between ACA- pSS and SSc.

Since Miyawaki et al [9] demonstrated that 6 out of 10 pSS patients with ACA and RP developed CREST syndrome in the follow-up investigation, it is necessary for us to follow-up the present 14 ACA+ pSS patients to determine whether they develop CREST syndrome in the future. Avouac J et al [16] also previously reported that the prevalence of secondary SS was 14% and was strongly associated with limited cutaneous SSc. Although we excluded limited cutaneous SSc in the present study according to the criteria determined by LeRoy et al [17], SSc complicated with secondary SS should be carefully examined during follow-up period. Since data from the above report [9] showed development of CREST syndrome in pSS patients with ACA, observation period of 6.6 years in this study might not be sufficient to refer to assess accurate follow-up period for development of CREST syndrome or limited SSc in the subset of ACA+ pSS patients. It is difficult to make mention of possibility for development to pSS or CREST syndrome/limited SSc unless a prospective study for ACA+ pSS subset is performed. Because this is an issue in the future, it might be difficult to cite from this study.

## Conclusions

In ACA+ SS, low cellular infiltration but with an increase in fibrous tissues may explain the clinical feature of a high prevalence of RP and normal serum IgG concentration in ACA+ pSS. These findings suggest that ACA+ pSS patients were different from patients with anti-SS-A/Ro and SS-B/La antibodies.

## List of abbreviations

Abs: antibodies; ACA: anti-centromere antibody; AECG: American-European Consensus group; APS: aminopropyltriethoxysilane; CCP: cyclic citrullinated peptide; ELISA: enzyme-linked immunosorbent assay; FS: focus score; MSG: minor salivary glands; pSS: primary Sjögren's syndrome; RP: Raynaud' s phenomenon; SSc: systemic sclerosis.

## Competing interests

The authors declare that they have no competing interests.

## Authors' contributions

HN participated in the design of this study and in collecting background data, performed image analysis, statistical analysis and drafted the manuscript. TH provided specimen material for Azan Mallory staining. AK, NI, AO, MT, SY, HI and EK performed critical reading for the manuscript. All authors read and approved the final manuscript.

## Pre-publication history

The pre-publication history for this paper can be accessed here:

http://www.biomedcentral.com/1471-2474/11/140/prepub
